# Coping with the Subjective Impact of COVID-19 among Older Adults in Puerto Rico

**DOI:** 10.1093/geroni/igab046.3680

**Published:** 2021-12-17

**Authors:** David Camacho, Yamile Marti, Sunghwan Cho, Thomas Buckley, Julia Vazquez, Denise Burnette, Humberto Fabelo

**Affiliations:** 1 University of Maryland Baltimore, Baltimore, Maryland, United States; 2 Columbia University, Columbia University School of Social Work, New York, United States; 3 VCU, Richmond, Virginia, United States; 4 Virginia Commonwealth University, NORFOLK, Virginia, United States; 5 University of Maryland, University of Maryland, Baltimore, Maryland, United States; 6 Virginia Commonwealth University, Virginia Commonwealth University, Virginia, United States

## Abstract

The COVID-19 pandemic poses serious physical and mental health risks for older adults worldwide. To develop culturally and contextually congruent services to mitigate these risks requires understanding their stress and coping processes, which remain understudied in Latin America. This study examines qualitative data from 51 adults aged 60 and over who participated in an ongoing study of older Puerto Ricans’ knowledge, attitudes, and practices about COVID-19. Trained interviewers collected the data by telephone from January to August, 2021. Two-thirds of participants were female, 60% had less than high school education and 90% had poverty-level incomes. Drawing on Lazarus and Folkman’s Stress and Coping Theory, we conducted a thematic analysis of responses to open-ended questions about the nature and extent of COVID-related stressors, stress management, and meanings and guidance they had gleaned from their experience. Participants perceived the pandemic as an added threat to ongoing chronic stressors (e.g., Hurricane Maria, poverty, political instability); disruptions in daily routines, family cohesion, and grief and loss processes; and increased isolation and loneliness. They reported using cognitive, behavioral, socioemotional and spiritual coping, including positive thinking, keeping occupied, relaxation, religious practices and, in a few cases, social media. Participants highlighted a revitalized appreciation for emotional qualities of relationships, freedom and life in general. Consistent with our guiding theory, cultural, contextual, religious, and socio-political factors shaped their appraisals of stress and their coping strategies. Future research should examine how these practices relate to health outcomes and quality of life and how they can inform effective, appropriate interventions.

